# Recognition of letters displayed as successive contour fragments

**DOI:** 10.3934/Neuroscience.2022028

**Published:** 2022-12-12

**Authors:** Sherry Zhang, Jack Morrison, Wei Wang, Ernest Greene

**Affiliations:** 1 Department of Psychology, University of Southern California, Los Angeles, CA 90007, United States of America; 2 Neuropsychology Foundation, Sun Valley, CA 91353, United States of America; 3 Departments of Medicine and Neurology, Brigham and Women's Hospital. Division of Sleep Medicine, Harvard Medical School, Boston, MA 02115, United States of America

**Keywords:** vision, persistence, letter encoding, masking, visual integration

## Abstract

Shapes can be displayed as parts but perceived as a whole through feedforward and feedback mechanisms in the visual system, though the exact spatiotemporal relationships for this process are still unclear. Our experiments examined the integration of letter fragments that were displayed as a rapid sequence. We examined the effects of timing and masking on integration, hypothesizing that increasing the timing interval between frames would impair recognition by disrupting contour linkage. We further used different mask types, a full-field pattern mask and a smaller strip mask, to examine the effects of global vs local masking on integration. We found that varying mask types and contrast produced a greater decline in recognition than was found when persistence or mask density was manipulated. The study supports prior work on letter recognition and provides greater insight into the spatiotemporal factors that contribute to the identification of shapes.

## 1. Introduction

Numerous studies have been conducted to answer the question of what features are needed for shape recognition and which processes may disrupt shape encoding mechanisms. Early research using dot arrays found that the visual system could integrate two partial images into a complete one [1],[2]. That research mainly used images and other visual tasks that did not require pre-learned knowledge, but similar studies have been done with full-letter perception [3],[4]. Letters have proven to be useful targets for examining visual mechanisms [5],[6]. Further work has been done by Greene and associates to examine the contribution of retinal and cortical persistence for the integration of partial shape cues [7],[8].

The present experiments cut outlined letters into a series of fragments, briefly displaying each adjacent fragment subset in sequence. The visual system must integrate the partial shape cues that are provided by successive fragments for the full letter to be identified. This has some similarities to early work where letters were displayed through a moving slit [9]. Rock and others describe this as “anorthoscopic perception,” and have suggested that physiological responses in both the retina and cortex play critical roles in the integration of the successive shape cues [10],[11]. Though the basic display conditions in our experiments may be similar to the moving slit work, we are more concerned with the changes in recognition when blank frames and masks have been added.

The first experiment displayed the fragment sequence across a range of contrast levels, using the probability of letter recognition as a function of contrast to establish the contrast sensitivity of each participant, as it can vary widely across individuals [12],[13]. In each of the experiments that followed, letter fragments were displayed at contrast levels that were determined by the contrast sensitivity of each participant. We used a target contrast that was just below reliable letter recognition to provide display conditions that were more sensitive to how time delays and masking would affect the perceptibility of the letters.

The second experiment added blank time frames between each fragment subset to monitor the contribution of stimulus persistence. Briefly displayed letter arrays persist for hundreds of milliseconds after the stimulus itself is no longer present and increasing the interval between partial shape cues has been shown to decrease recognition [3],[14],[15]. The rate of recognition decline can best be explained by invoking cortical persistence mechanisms. It has been shown that contour fragments are registered by orientation-selective neurons in V1 [16]–[19]. Our working hypothesis is that our letter fragments would be registered by these orientation-specific neurons, and the linkage of activity among these neurons would provide a more complete, identifiable letter [20],[21]. Because the activity of the V1 neurons is relatively brief, we expected to see a decline in recognition when blank trials increased the interval between successive letter fragments.

Our third experiment inserted a full-field mask consisting of partial letter fragments whose density was varied. This follows work that evaluated the effectiveness of pattern masks on recognition [22]–[24]. It is likely that masking disrupts comprehensive signals from V1 to the rest of the brain [25]–[27]. We further hypothesized that increasing the power of masking through density would create a monotonic decline in recognition due to the increased number of other elements in each mask frame. We predicted that increasing density would thus create a more powerful mask.

The final experiment used mask strips that were briefly displayed, successive strips falling across locations at which the letter fragments had been shown. The contrast of the masks was varied, with the expectation that the masks would become progressively more effective as they reached or exceeded the contrast of the letter fragments. This method provided for close spatial and temporal action of the mask on elemental shape cues. Our work is similar to prior metacontrast studies that have used non-overlapping masks to impair target recognition [24],[28]–[30]. Related work on the effect of stimulus crowding and contrast suggests that the contrast of masking would have significant effects in impairing recognition [31].

## 2. Methods

### 2.1. Institutional Approval and Participants

Research protocols were approved by the USC Institutional Review Board. Forty-five USC undergraduates (34 females; nine males) served as participants, each providing data for each of the four experiments for course credit. Ages ranged from 18 to 26. The nature of the task was explained to each participant, but no feedback on performance was provided during the experiments.

### 2.2. Letter and Mask Displays

The 26 capital letters of the English alphabet were displayed on the computer monitor of each participant. Each letter was half the height of the full screen, providing a mean height of 12.2 degrees of visual angle. Each letter was defined with white contours that outlined the strokes, against a black background that filled the screen. Pixel intensities of a monitor can range from 0 (off/black) to 255 (maximum brightness), and for all experiments, the application was able to specify the contrast of the contours on any given trial in one-unit increments.

Each letter was displayed as fragment subsets that were derived by cutting the screen image into 20 strips. The width of strips depended on the randomly chosen orientation and direction of successive strip sequences. The number of strips that would contain letter fragments depended on the orientation that had been chosen for a given letter display and the width of the letter. Averaging across all these conditions, the mean number of strips that would contain letter fragments was 5.6, with the range being 2 to 12 strips. Median and mode were both found to be 6 strips, with 79% of the letters falling in the range from 4 to 6 strips

**Figure 1.**
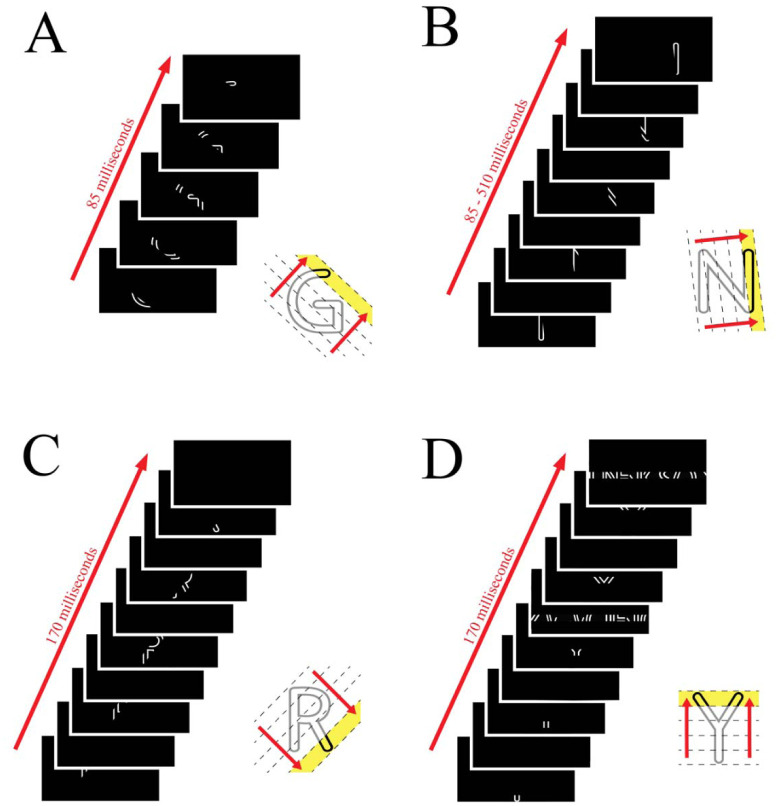
The letter display for each of the four experiments is illustrated. Letter fragments were displayed in successive 17 ms time frames. The number of fragment subsets depended on the letter being displayed and the orientation of the sampling strip, five being provided here for purposes of illustration. The four illustrations show letters displayed at different orientations under different conditions. The first shows a letter with no blank frames in between fragments. The second and third show letters with varying amounts of blank frames in between each letter fragment. The fourth shows both the letter fragments and the frames of nonsense letter strips that were used as a mask.

An LCD display updates images once every 16.7 ms (hereafter designated as 17 ms), which is designated as a “time frame.” Letter fragments in spatially contiguous strips were displayed in successive time frames, as illustrated in Fig 1A. The average time for display of the full sequence was 102 ms (6 frames x 17 ms per frame).

Masking stimuli were constructed by cutting letters into four pieces, designated as “mask fragments,” and then providing an array of randomly selected mask fragments. A different mask pattern was used for every trial in which a mask treatment was provided. The density of a mask pattern was controlled by adjusting the uniform spacing (possibly overlapping) between mask fragmnts. For example, a mask density of 1.0 was achieved using spacing such that the mask pixels covered 9.7% of the display area, where 9.7% is the average coverage of the letter pixels. A mask density used a smaller spacing, e.g., with density = 2.0 provided 19.4% of pixels displayed per unit area.

### 2.3. Common Experimental Protocols

In each of the experiments, letters were selected at random for each trial. Each trial began with a blank (black) screen that had a small white plus sign as a fixation point at the center of the screen. Letters were displayed upright (without tilts) at eccentric screen locations to inhibit recognition based on distinctive location attributes, e.g., preventing vertical contours from being reliably at the center for an I or T, or to the left of the center for an L. For this purpose, the letter was displayed offset by half of the letter height at an angle that was randomly selected for each trail. The orientation of the strips was varied at random from one trial to the next—some being vertical, some horizontal, and others being at various diagonal orientations. A given strip sequence could begin at any location, chosen at random, around the perimeter of the screen.

The experiments were done remotely for each participant on their individual computers. Participants were asked as part of the instructions to keep their computers a consistent brightness for all parts of the experiment. Participants were further asked to do no more than one experiment a day. Each participant was tested with an online protocol, first being given instructions for how to download a custom application that was designed either for a Mac or Windows operating system. At the launch of the first experiment, they were provided with a questionnaire to gather data that might be used as covariables. This included screen dimensions as well as a measure of viewing distance, those values being needed to compensate for letter size (see *Covariates*, below). Age, gender, computer type, letter size, and resolution were analyzed as covariables for additional controls and are discussed in more detail in the *Covariates Analysis* section.

After the launch of a given experiment, the participant would press the space bar of the computer to begin a trial, which caused the fixation point to disappear and launched the strip sequence. A keyboard response was used to record whether the participant recognized the letter, but the participant was required to guess even if the letter was not identified by choosing one of the letter keys. The computer recorded the keystroke along with the treatment variables for that trial. After a letter keystroke was executed, the blank screen with the fixation point would re-appear, ready to provide the next letter display when the space bar was pressed again.

After all trials of a given experiment were run, a data log appeared on the participant's computer. The custom application was automatically disabled to assure that the data came from one and only one test session. The participant then emailed the data log to the experimenters. After the log for a given experiment was received, a link that allowed the download of the next experiment was sent to the participant. This process was repeated until data from all four experiments had been returned.

### 2.4. Specific Experimental Protocols

Experiment 1 was designed to determine the frequency of recognition as a function of letter contrast, i.e., the contrast of the letter fragments. This was done in two sessions. The first session, designated as Ex 1.1, varied letter contrast across seven levels: 10, 15, 20, 25, 30, 35, 40. The contrast level to be used was varied at random for each trial, requiring also that each level be used for the display of 20 letters, for a total of 140 trials.

A second session, designated as Ex 1.2, used the same protocols but modified the contrast range for many of the participants. In the first session, some participants were well above chance levels of recognition even at the lowest contrast level, i.e., 10. Exp 1.2 provided these participants with a lower range: 0, 5, 10, 15, 20, 25, 30. Other participants didn't reach 100% recognition in Exp 1.1 at the highest contrast level, i.e., 40. Exp 1.2 tested these with 25, 30, 35, 40, 45, 50, and 55. In all cases, 20 trials were provided at each contrast level for a total of 140 trials.

The data from Ex 1.1 and Ex 1.2 were combined to provide the frequency of recognition plots for each participant. Using these plots, we determined the contrast that would be expected to yield a probability of recognition (hit rate) of 85% for each individual participant. This is designated as Lc85% (individual contrast probability for 85% recognition). For each participant, their LcP85 level was used for the display of letters in each of the following three experiments.

Experiment 2 displayed the letter fragments to each participant using the Lc85 that had been derived from Ex 1. Each trial added time frames displaying the black background between successive letter fragments, the goal being to extend the interval and thus impair the integration of shape cues. The number of “blank” time frames varied from 0 to 6, each being displayed for 20 trials (140 total trials).

Experiment 3 displayed the basic letter-strip sequence alternating with time frames that displayed a full-field mask pattern. Mask patterns were displayed at the same contrast as the letters, i.e., at the Lc85 contrast level. The density of mask patterns were scaled based on the average number of contour pixels per unit area, as specified above. Mean density of letters was normalized to 1.0, and the density of the mask patterns were multiples of this value. Six levels of density were provided -- D = 0X, 0.5X, 1X, 1.5X, 2X, 2.5X. Twenty letters were displayed at each density level, for a total of 120 trials.

Experiment 4 provided sequences of letter fragments alternating with mask strips, displaying letter fragments at the Lc85 contrast level. All mask patterns used a 1X density, i.e., the same density as letters, with a different pattern being constructed for each trial of the experiment. Mask contrast was varied across four levels that were multiples of the Lc85: 0X, 1X, 2X, 3X. Thirty trials were provided at each contrast level for a total of 120 trials.

### 2.5. Covariates

Participant logs contained records of ancillary conditions that might affect performance. This included the age and gender of the participants (reported above). All but nine of the participants used Mac computers, and all but one of these were laptops. Participants measured and reported the dimensions of the screen and the viewing distance. The letters themselves were half the height of the screen, so these measures allowed the height to be specified as a visual angle, providing a potentially useful covariable. The app itself was able to extract a record of screen resolution (pixel density) across the width and height for each of the participants, again providing potential covariables.

For any of the covariates that were significant, or marginally significant, we thought it would be useful to determine the size of those effects relative to the main treatment variable (letter contrast). Effect size is not well defined for logistic models, but from the ROC curves, one can derive an AUC (area under the curve) statistic that is commonly accepted as reflecting effect size. AUC values range from 0.5 to 1.0, with the latter value indicating perfect classification. The AUC value where only the letter contrast was provided equaled 0.9288. Gender added only 0.0008 to the 0.9288 AUC value, thus contributing very little to overall treatment effects. The combination of X screen resolution plus the interaction term produced a change in letter recognition of 0.0045. The values for Y resolution were virtually identical to X resolution. It seems clear, therefore, that letter contrast was the dominant factor controlling the recognition of letters, so we will have little more to say about the covariates in the discussion that follows.

### 2.6. Statistical Methods

Mixed-effects logistic regression models were applied to study the association between the probability of recognition and experiment conditions (letter contrast in Experiment 1, the number of blank time frames in Experiment 2, mask density in Experiment 3, and mask contrast in Experiment 4). Participants were treated as random effects. Potential covariates such as age, gender, computer type, horizontal (X) and vertical (Y) screen resolution and letter height were tested in the model for their contribution to the probability of recognition. The area under the receiver operating characteristic curve statistics were calculated and compared between models with and without covariates to assess the effect size of these covariates. The level of significance for all analyses was set at 0.05. All data analyses were conducted using SAS 9.4.

## 3. Results

We conducted four experiments to clarify the nature of contour encoding, focusing especially on the time required for shape cues to be integrated and disruptions of recognition that could be produced by masking. Letters were used for the present work, as their overlearned nature provides more reliable measures of recognition probability. Each of the 45 participants was tested in each of the four experiments, and all analysis was based on the full complement of data.

### 3.1. Covariate Analysis

We documented a number of factors that might affect perception and/or memory retrieval. Age was considered, as many visual skills are known to decrease with age [32]–[34]. We found no significant age differences across any of the four experiments, but this might well be due to the highly restricted age range—all of the participants were university undergraduate students that were similar in age.

Gender was of interest as prior work had indicated that it produced visual perception and visual-spatial performance differences [35]. However, significant gender effects were not manifested in any of the first three experiments. A probability of p = 0.06 was found in Exp 4 where the interaction term was included in the analysis, which might be considered to be marginally significant. However, the effect size of this variable was minimal, as further elaborated below.

Computer type and letter size did not make significant contributions to letter recognition in any of the four experiments. Here again, the restricted range of alternative conditions could readily explain why one would not find a difference for computer type. Letter size, however, ranged from 5.2 to 25.1 degrees of visual angle. It is a testimony to the effectiveness of size invariance mechanisms that the differences in letter size did not affect recognition.

The role of screen resolution and their effects on visual perception have been studied in both fields of vision as well as engineering [36],[37]. For the present work, both X and Y screen resolution proved to be significant in Exp 1 but not in any of the other three experiments.

**Table 1.** Table cells show the error probabilities for testing whether a given covariate contributed to letter recognition in addition to experimental conditions in the mixed-effects logistic regression models. The area under the receiver operating characteristic curve statistics were calculated and compared between models with and without covariates to assess the effect size of these covariates. The level of significance for all analyses was set at 0.05 Across the four experiments, only X and Y screen resolutions (pixel density) proved to have a significant influence, and then only in Exp 1. Though significant, the size of their contribution to letter recognition was extremely small in relation to the main treatment effect of letter contrast.

	Age	Gender	Mac/Windows	Letter Size	X Resolution	Y Resolution
Exp 1	0.21	0.83	0.44	0.35	0.0001	0.0001
Exp 2	0.19	0.34	0.84	0.25	0.81	0.98
Exp 3	0.99	0.83	0.38	0.76	0.29	0.37
Exp 4	0.27	0.06	0.66	0.15	0.46	0.51

### 3.2. Experiment 1: Contrast and recognition

**Figure 2.**
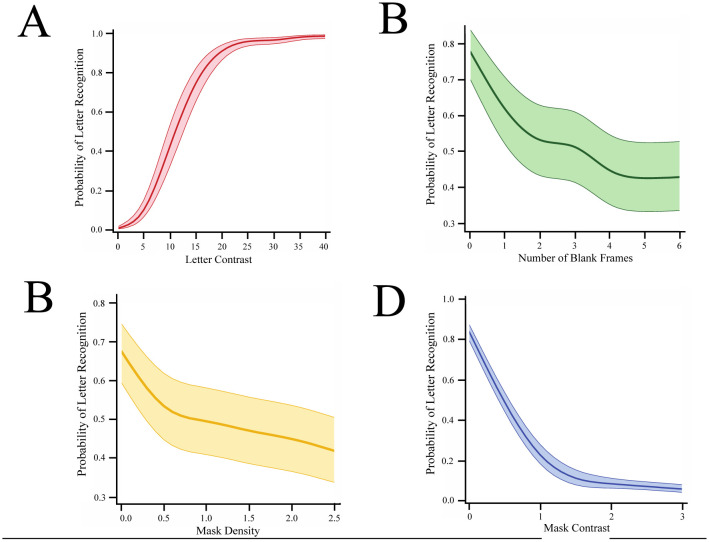
Logistic regression models were derived for each of the four experiments. A. Exp 1 varied the size of letter contrast, and a probability of letter recognition function that increased from chance levels to reliable identification of letters was derived for each of the 45 participants, the group model is provided here. B. In Exp 2, the probability of recognition manifested a monotonic decline as a function of the number of blank frames that were displayed between letter fragments. We interpret this as a decline in the persistence of the shape cues provided by the successive fragment subsets. C. In Exp 3, an increase in the density of mask components produced a monotonic decline in the probability of letter recognition. D. In Exp 4, the display of mask strips that overlapped the location of successive letter fragments provided very effective masking of letter recognition. The contrast level of the mask strips was a major factor in the impairment of letter recognition, as reflected in the group model shown here.

The first experiment used the display sequence that is illustrated in Figure 1A. It assessed how increasing levels of letter contrast provided for the progressively higher probability of letter recognition, as shown by the logistic regression model in Figure 2A. This result is consistent with prior work that used low-contrast stimuli [38],[39]. The letter contrast that was displayed on a given trial was chosen at random within the range of levels being provided to each participant. Judgments for this experiment were collected over two sessions with the regression being calculated over the mean of the two sessions.

The rise in the probability of recognition (hit rate) as a function of letter contrast was significant at p < 0.0001. Recognition was significantly different than chance at the lowest level of letter contrast, in that the 5 contrast produced a mean hit rate of 9.67%, which differs from a chance rate of roughly 4% with p < 0.0007. A plot of the mean hit rate for the group of 45 participants as a function of letter contrast is provided in Figure 2A, which includes the 95% confidence band for the group.

### 3.3. Experiment 2: Blank frames manipulation

The second experiment displayed letter fragments at each participant's Lc85 and provided a variable number of blank (black) time frames between each time frame that displayed letter fragments (see Figure 1B). The logistic regression model for these data is shown in Figure 2B. Recognition was just a bit below the expected 85% hit rate where the display sequence contained no blank time frames. Letter recognition declined dramatically with the addition of a single blank (17 ms) time frame between each fragment subset, and it continued to drop as more blank time frames were inserted. The decline in recognition is likely due to decay to the persistence of cue information.

Regression for the probability of recognition as a function of the number of blank time frames was significant (p < 0.0001), with insertion of even a single blank time frame providing a decrease in hit rate that was significant (p < 0.0001). The confidence interval is substantially wider than was found in the first experiment, reflecting the fact that the recognition performance of participants differed far more for these display conditions. None of the covariates made even a modest contribution to performance (see Table 1), so the increased variability likely reflects intrinsic individual differences in the ability to integrate (link) letter fragments across an interstimulus interval.

### 3.4. Experiment 3: Full-field masks and mask density manipulation

The third experiment examined the impairment of recognition that was produced by inserting a full-field mask pattern between each time frame that displayed letter fragments, i.e., alternating letter fragments and mask pattern displays (see Figure 1C). Recognition of letters declined as a function of mask density, as can be seen in the regression model (Figure 2C). The decline was significant (p < 0.0001) with a significant impairment being produced when the mask pattern was only half the density of the letters (p > 0.0001). The wide confidence band again reflects the wide individual variability of performance by participants on this task.

### 3.5. Experiment 4: Strip masks and mask contrast manipulation

In Exp 4, the display of each letter-fragment subset was followed in the next time frame by a mask strip that spatially overlapped the location of the fragments (see Figure 1D). The regression model in Figure 2D shows the decline in recognition that was produced by this masking condition. The decline was significant at p < 0.0001. Mean recognition was roughly 80% where a blank time frame was presented instead of a mask, which dropped to just above 20% where the contrast of the mask strips was the same as letter contrast. This also was significant at p < 0.0001. Increasing the contrast of the mask strips produced further impairment of recognition, dropping recognition to just above the chance range when the mask contrast was three-times greater than letter contrast. For this experiment, the variability among participants was relatively small, as it was for the first experiment.

### 3.6. Comparable Treatments Across Experiments

Experiments 2, 3, and 4 each had a main treatment that was manipulated, i.e., providing various quantitative levels of the treatment. However, there were a few instances where a given combination of treatment levels were the same across experiments. The following comparisons are for specific treatment combinations that displayed the same letter fragments, blank time frame, and or mask. These were the combinations where one might expect letter recognition to be the same.

**Figure 3.**
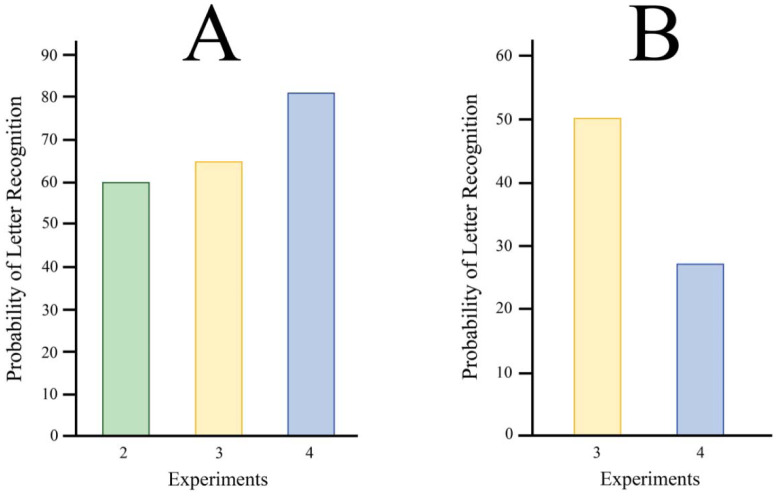
Across experiments there were differentials in probability of recognition for treatment conditions that were identical. (A) Level 1 of Exp 2 added a blank time frame between each time frame displaying letter fragments. (B) The 1X conditions of Exp 3 and Exp 4 provided masks having density equal to 1 and contrast equal to 1. The experiments differed in that the former provided a full-field mask between each letter-fragment time frame and the latter provided strips of mask pattern that overlapped the letter fragments.

Exp 2 added a blank (black background) time frame between each time frame displaying a subset of letter fragments. This extended the interval between successive fragment subsets, interfering with the integration process, and reducing recognition from the 85% hit rate that would be expected when no separation was present (as in Exp 1). A similar reduction was seen in Exp 3, wherein the 0X condition provided a blank time frame between each fragment-subset, in that the density of the mask for this condition was zero. Display conditions were the same for the 0X level of Exp 4, where the contrast of the mask was zero (no mask shown). Yet there were differentials in hit rate even though the experimental treatments were the same, as reflected in the bar graph provided in Figure 3A.

The difference in Exp 2 vs. Exp 3 was not very large, but it did prove to be significant at p < 0.018. And the impairment of recognition in Exp 4 relative to the other two experiments were each significant at p < 0.0001. The difference might be attributed to practice effects, which have been shown to increase visual acuity over time [40]. Follow-up experiments could be done in random order to determine the effect of practice.

In Experiments 3 and 4, mask density and intensity were the same for the 1X condition in both experiments. The 1X conditions of Exp 3 and Exp 4 provided masks at a density equal to 1 and contrast equal to 1. Yet this combination of conditions produced a substantially greater decline in letter recognition in Exp 4, as reflected in the bar heights of Figure 3B. That difference in hit rate was significant at p < 0.0001. The experiments differed in that the former provided a full-field mask between each letter-fragment time frame and the latter provided strips of mask pattern that overlapped the letter fragments, suggesting that the difference came from the effectiveness of the mask type. The strip sequence was substantially more effective at disrupting letter recognition.

## 4. Discussion and conclusion

The basic goal for this line of research was to provide display conditions that controlled the perceptibility of images and provided for retrieval of shape information from memory. Effective research protocols should deliver cues that are essential for shape recognition and control for those that are less relevant. It was gratifying, therefore, to find that the variables chosen for manipulation provided, by far, the greatest influence on letter recognition. The various levels of letter contrast (Exp 1) provided dramatic control of perceptibility, yielding probabilities of recognition that rose from chance performance to reliable identification of each letter that was displayed. Modifying the spacing between letter fragments (Exp 2) impaired letter recognition as a function of the temporal separation of the fragments. This addressed how the integration of local contour segments can provide a perception of intact contours that define a given letter. Adding a full-field mask pattern between each subset of letter fragments (Exp 3) disrupted the retrieval of information from memory, as did superimposing a mask strip on each of the successive fragment subsets (Exp 4). The present research asked for the recognition of letters, but clearly, these display conditions and treatments have the potential to clarify the general nature of shape encoding and shape recognition. Therefore, the following discourse will discuss shape encoding and retrieval mechanisms and will use the terms letter cues and shape cues interchangeably.

### 4.1. Segmentation and Sequential Display of Letter Contours

Outline contours were the shape cues in the present work, and each letter provided a distinctive configuration of stroke outlines. It is well established that the primary visual cortex has neurons that are designed to register these contours [16]–[19], with a given neuron being especially responsive to a contour that lies at the orientation of its elongated “receptive field.” What is less well appreciated is the fact that the receptive fields of V1 neurons are relatively short, with the average length being about one degree of visual angle and with the longest being about two degrees [41]. For most shapes that we can identify, the contours that define the shape may be much longer than this, so the full contour that we think of as providing a distinctive shape cue may have to activate a number of V1 neurons. Each of the experiments sliced the outline contours of the letters into contour fragments and displayed these fragments sequentially. The average height of the letters was just over twelve degrees of visual angle, so the typical length of vertical fragments would be about two degrees of visual angle. This would correspond to the length of the largest receptive fields of orientation-selective neurons in the primary visual cortex. To identify a given letter, the visual system had to combine the sequential cues provided by the fragments.

Numerous investigators have hypothesized that the degree of stimulus simultaneity is a factor in binding together common features in an image [42]–[44]. The stimulus binding would likely be reflected in synchronized firing of cortical neurons. Many labs have demonstrated that these neurons can synchronize with precision in the millisecond range [44]–[46]. Precise synchronization of spatially separate neurons has been demonstrated in the striate and extrastriate cortex of primates [47],[48]. While most of this work has documented the linkage of neuron activity with millisecond differentials of stimulus display, it is possible for entrained oscillation to provide for long-range linkage as well as synchronization of activity over extended time intervals [49],[50]. Experiment 1, therefore, provided the base condition for further study of how increased temporal separation of fragment subsets, and how the inclusion of mask stimuli, would impair contour integration.

### 4.2. The Role of Persistence in the Integration of Shape Cues

There was a significant decrease in recognition of letters as the time between successive fragment subsets was increased in the second experiment. This likely was due to a decay in visual persistence that mediates integration of the fragment cues, resulting in impaired recognition of the full letter. By using a target contrast that was just below reliable letter recognition (Lc85), we provided display conditions that were sensitive to additional time delays. A higher contrast would likely extend the duration (strength) of persistence, maintaining recognition at 100% even with inclusion of one or more blank time frames. Displaying the fragments at the Lc85 contrast increased the sensitivity of the task to the inclusion of time delays. Prior work on the phenomenon of inverse-persistence and inverse-duration effect would predict that the higher contrast mask would be less effective [51],[52].

Prior experiments from this laboratory have examined the role of persistence. Greene and Visani studied the recognition of letters that were displayed as patterns of flashed dots within an array of LEDs [7]. The experiments that were most comparable to the present work displayed two sparse, non-overlapping patterns, neither yielding much letter recognition when presented alone, following the paradigm of Eriksen & Collins [3]. Recognition was less than 40% if only one of the patterns was displayed. The hit rate was 85% if both patterns were displayed at the same moment. But the insertion of an interval between each pattern impaired recognition, reaching the one-pattern level in about two-hundred milliseconds. Greene followed up with similar experiments wherein the letter strokes were represented by very sparse patterns of aligned dots [53]. Again, two non-overlapping subsets were displayed with progressively longer temporal separation, and with these stimulus conditions, the decline in recognition reached the one-subset range in about six-hundred milliseconds.

In these earlier experiments, the shape information persisted for two- to six-hundred milliseconds, both intervals being long enough to implicate the role of working memory or a short-term visual memory, which is theorized to protect against masking [54],[55]. In the present work, each fragment subset was displayed within a seventeen-millisecond time frame. Across letters, the average number of time frames displaying fragments subsets was just under six, so the display of a given letter with no additional time frames added was about a hundred milliseconds. Adding blank time frames increased the display time, and the second experiment found that recognition appeared to be reaching an asymptote with the addition of four blank time frames, i.e., an interval of roughly four-hundred milliseconds. Therefore, the present findings are within the range of persistence effects reported by Visani & Greene (2015) and Greene (2016), this being an interval within which one would expect a role for working memory mechanisms [7],[53]. The cue integration that is needed for shape recognition may well be taking place through cooperative activation of orientation-selective neurons in V1, possibly assisted by feedback from memory.

The question of what neuron populations are involved in registering and integrating the contour fragments is of special interest. The shape cues provided by these fragments can reach cortical systems very quickly. Bair and associates found that signals can pass from the retina to the lateral geniculate nucleus in macaque within fifteen to twenty milliseconds and enter the primary visual cortex (V1) in twenty to twenty-five milliseconds [56]. Orientation-selective responses are manifested among V1 neurons within fifty milliseconds after retinal stimulation [57],[58]. Further, the shape cues are able to pass into extrastriate populations with minimal delay. Vogels recorded spike activity in the anterior temporal cortex of macaque and found selectivity of response to displayed shapes within eighty to ninety milliseconds after stimulus presentation [59]. Similar fast responses have been shown to other stimuli, including words [60].

The work cited above speaks to the speed at which shape cues can be passed to the brain populations that mediate the perception and recognition of shapes. However, those studies provided a simultaneous display of a complement of cues that would be sufficient for recognition. The more important consideration here is the degree to which persistence of the cue information would allow integration across a sequence of fragmentary cues, as shown in early work where full letters could be identified when shown as fragments through a slit [9]. Classic work into anorthoscopic perception has suggested there is a interplay of physiological responses in the retina and processing in other areas. While the initial presentation of letters in our experiment may be similar to these studies, we are more concerned with the changes in accuracy when blank frames and masks were added.

We assume that the fragments provided within a given time frame are being registered by the orientation-selective cells in V1. It is less clear how integration across fragments provides for the perception of the full letter. One issue is the transience of response to a brief stimulus display. Firing that lasts longer than the stimulus has not been observed in the orientation-selective neurons of V1 [16],[61],[62]. The lack of activity cannot be attributed to anesthesia because the same results have been found in behaving animals [18]. In fact, more often the firing of V1 neurons is shorter than the duration of the stimulus itself [63],[64].

There is evidence, however, that neuromodulation may provide for population activity in V1 that is not reflected in the sustained spiking activity of individual neurons. Short-term synaptic plasticity over roughly minutes might be provided by transmitter-mediated neuromodulation [65],[66]. Nikolic and associates studied distributed responses in a population of V1 neurons as successive letters were displayed [67]. Recorded activity from a population of neurons reflected which letter had been shown, and that activity was manifested for periods that ranged from four hundred to seven hundred milliseconds.

Castelo-Branco and associates (1998) reported that flashed bars or gratings triggered high-frequency (60–120 Hz) gamma oscillations in the retina and lateral geniculate nucleus that lasted for about a second [68]. Fries and associates recorded local field potentials from a population of orientation-selective cells in V1, finding that the integration of letter fragments produced gamma waves in this population [20]. They proposed that gamma waves were needed for the synchronization of adjacent receptive fields. Moving bars of light produced synchronization of gamma waves associated with the oscillatory firing of neurons over long distances, such as across the striate cortex and between hemispheres [49]. These results suggest that higher-level visual integration needed for recognition begins with orientation-selective cells and is mediated by synchronized gamma waves. Based on the time interval across which these waves were seen, we predicted that mediation would fail over longer periods of time between frames, thus lowering recognition as blank frames were added. Our results were supported, though recognition never fell to the chance level.

By contrast with the rapid termination of firing by neurons in V1, cells in the inferotemporal cortex can manifest sustained firing for several hundred milliseconds [69],[70]. This appears to be especially true if the animal is required to remember information from an earlier period [63],[71]. Keysers and associates provide especially compelling evidence that neurons in the inferotemporal cortex and within the superior temporal sulcus are involved in integrating successive image cues [72].

An alternative index of message delivery comes from research done with event-related potentials. Recent EEG research supports the idea that event-related potential (ERP) components are activated during object recognition within roughly one-hundred-fifty to three-hundred milliseconds of stimulus display [21]. These ERPs have been theorized to play a role in top-down processing from higher cortical areas [26]. Other research has found that ERPs within the occipitotemporal cortex may be necessary for delivering spatiotemporal information when words or letters are read [73]. Along the lines of these studies, we predicted that our experiments would interfere with this ERP, causing difficulty in recognition and lowered accuracy across the trials.

One may also consider higher-level structures and their role in global shape encoding. Orlov and Zohary have suggested that the lateral occipital complex is responsible for the global encoding of shapes [11]. This study used fMRI and multivoxel pattern analysis to examine the brain regions involved in the integration of shape from parts. Participants were shown images through stationary slits and judged their similarity to full images. Researchers found similar activation patterns in the lateral occipital complex when shapes were seen behind a slit as when the full image was judged, suggesting that this brain area mediates the temporal integration of segmented images. Though our experiment had participants integrate letter fragments over across a screen rather than through one slit, it is possible that the lateral occipital cortex played a similar role.

Our initial hypothesis was that increasing the interval between successive time frames would impair the linkage of fragments by orientation-selective neurons in V1. Taking into consideration the complex communications among different brain regions, as discussed above, the increase in delay is shown to extend past the timing required for these interactions. The results are consistent with a revised hypothesis that persistence of extrastriate activity mediates the assembly of a complete picture from incomplete fragments. The significant decrease in hit rate with increased delay shows that when fragments can't be held for an extended time through the persistence, integration and comprehension of the full letter fails.

### 4.3. Full-field Masking Effects

Early research has shown the effectiveness of masking on recognition impairment [24],[43],[74],[75]. We hypothesized that increasing the density would lead to a decline in the recognition of letters and results from the third experiment supported this hypothesis. The third experiment found that recognition decreased as the density of a full-field mask was increased. A simple explanation of density effects on recognition may be that increased density induces camouflage effects of the mask on the target letter, making them harder to locate. An increase in density raises the number of edge-intersections, and this kind of manipulation has been shown to reduce the likelihood of stimulus detection [76].

Interruption of cue processing may be relevant, in that prior work has shown that masking is more effective when presented in close temporal relation to target stimuli [24],[74],[75]. Masking was only effective at interrupting recognition when it was displayed within 100 milliseconds of the target. Rather than providing camouflage, the mask strips may be quenching the persistence of fragmentary cues, thus preventing integration across the sequence of cues. This interruption has further been studied in the context of visual cortex activity in order to understand the mechanisms by which signals are interrupted.

In a texture discrimination study by Lamme and associates, they found that displaying a texture mask within one-hundred milliseconds of the texture target decreased the visibility of the target and suppressed V1 activation, leading to impaired stimulus detection via interrupted figure-ground segregation [77]. However, the study found that response by orientation-selective neurons was not entirely eliminated, allowing for some integration of the target stimulus. For the present stimulus conditions, it is possible that orientation-selective neurons were integrating a few sets of contour fragments, allowing them to come into full awareness. Integration of top-down influences from higher cortical areas and feedforward effects from visual areas may play a collaborative role in recognition.

Masking can provide EEG correlates that reflect the degree of visual awareness, possibly related to locating the letter fragments on the screen. When participants were shown brief images in between patterned full-field masks, they manifested a negative ERP on trials where they could discern the image, but not those in which the images were barely recognizable [78]. Our results support the idea that conscious awareness is necessary for the recognition, even when the stimulus is overlearned, and that masking disrupts this awareness. The ERP was seen around 260 milliseconds after the display of the mask and provided a consistent marker for awareness. Though other studies have indicated that a good deal of subliminal processing can occur in the occipitotemporal pathway within ~250 milliseconds [79], it is possible that conscious awareness is needed for full letter integration.

Masking decreases the size of ERPs that are generated one-hundred-fifty to two-hundred milliseconds after the presentation of the stimulus [26],[27]. Fahrenfort and associates report that masking does not disrupt early onset ERPs, i.e., those produced in less than a hundred milliseconds [25]. Based on this finding, they suggest that masking disrupts top-down rather than feed-forward processing. This hypothesis might apply to the present findings. It is possible that masking is not disrupting the linkage of letter parts, but rather is acting on brain areas that disentangle the combination of mask and letter.

The time frame of this early negative ERP may indicate an upper range of how long it takes for visual awareness of a stimulus to enter consciousness. It might be generated by activating the posterior inferior temporal gyrus, a region that is thought to be necessary for word recognition. Dien and associates provided fMRI evidence that the conditions that evoke this negative ERP also evoke reactions in the posterior inferior temporal gyrus [80]. Our study looked at recognition of letters rather than full words, but it is possible that activity in this brain region is also needed for letter recognition.

Other masking studies have found that the left occipitotemporal pathway has a role in binding letters into words [81]. Using fMRI, Dehaene and associates found that word processing became increasingly invariant as the activity progressed into higher cortical areas. The left occipital visual word-form area has been shown to be hierarchically organized, becoming more sensitive to higher-level components as words were processed [82]. Feedback from these word-processing areas may be playing a key role in letter recognition within our experiment.

The nature of our experiment has provided a way to assess global masking in the context of integrating multiple spatially and temporally separate shape cues. We hypothesized that increasing the density of the masking would increase the effectiveness of the mask due to an increase in camouflage elements on the screen. The display of mask patterns in every other time frame likely generated integrated masks that served as background. Our findings are consistent with theories of how camouflage impairs visual processing. Further, the results show that masking can impair the integration of fragmented shape cues, not just the recognition of an intact shape.

### 4.4. Spatially Targeted Masking of Fragments

The fourth experiment found that: a) superimposing a strip of the mask pattern on each successive set of letter fragments is very effective at disrupting integration; b) the mask pattern becomes especially effective when its contrast matches or exceeds that being used for the letters. We find it interesting that the most dramatic decline in recognition was found with these display conditions. The effectiveness of masking stimuli in smaller fragments, rather than a full-field mask, has been shown in many metacontrast and paracontrast studies [28],[29],[83]. The fourth experiment minimalizes the differences between para and metacontrast masking using multiple target letter fragments and multiple mask fragments between each target. Further discussion of this unique masking will be discussed. The use of multiple mask strips in between letter fragments may provide additional challenges to the recognition that will require further study.

The large difference in the probability of recognition in the fourth experiment versus that of the third experiment may be explained by the difference in spatial consistency of the two mask types. Although participants did not see a perfectly fused background in the third experiment, display of the same pattern in every other time frame provided spatiotemporal stability for the mask that was far greater than the sequence of fragment subsets, which displayed at different locations. In contrast, in the fourth experiment, both the mask strips and the letter fragments had transient spatial components as the sequence shifted to different locations across the screen. The spatial consistency of the full-field mask may have allowed for better figure-background segregation than was provided by mask strips. Studies have shown that figure-ground segregation is important for recognition of stimuli [77], and it is likely that a strip sequence would not be registered as a proper “background”. It is unlikely that a mask pattern that was presented as successive strips would be perceived as background. However, when a given strip falls across the locations of a set of letter fragments, it is very effective at masking perception of the fragments.

The importance of the contrast of stimulus and mask deserves additional comment. Early work by Michaels & Turvey (1979) showed differential effects of mask contrast on recognition using dark masks [84]. Their results showed that masks with a higher contrast were overall more effective, regardless of letter shade, similar to the monotonic decrease in accuracy found in Experiment 4.

Pelli and associates have shown that increasing the contrast of masks leads to reduced recognition of target letters when using non-target letters as metacontrast masking [31]. As the contrast of non-target letters was increased, participants took longer to identify the target letter. While it is possible that the mask strips were acting as distractors, it is more likely that the mask strips were disrupting integration processes shown by the drastic decrease in accuracy at high contrasts. Furthermore, due to the dissimilarity between each mask strip and letter, it is unlikely that participants mistook the mask fragments to be part of the target letter itself.

One should consider the potential role of visual short-term memory, i.e., the ability to store a limited amount of visual information for recall. Sligte and associates showed that pattern masking heavily disrupted visual short-term memory and subsequent recognition [85]. They had participants go through a change detection task with randomly oriented lines and used different masks to disrupt recognition. They found that a random-pattern mask completely overwrote visual short-term memory in their experiment, leading to significantly lower figure recognition. It is possible that the masking with strips of random letter components may be disrupting visual short-term memory in a similar fashion, intruding upon a fragile perceptual state, and not allowing further integration of shape cues.

One must also consider the possibility that the impaired recognition that is produced by mask strips may be from attention shifts rather than impaired contour integration. Metacontrast masking experiments done by Enns and DiLollo showed that a visible stimulus can be rendered invisible by rapid presentation of a mask that surrounds the target stimulus [29]. Enns and DiLollo theorized that masking primarily occurred because of a mismatch between top-down and bottom-up processes, leaving an unrecognizable message for the visual system [29]. It is possible that top-down processes are expecting integratable letters, but instead are provided with nonsense mask strips. This might rapidly shift attention from fragment subsets to the mask-strip pattern, impairing recognition in a way that is different than the influence of full-field masking.

Another explanation could involve the interference of multiple paths of integration within the brain. Working with mice, Meneghetti and associates highlighted the presence of broad and narrow gamma bands in V1 that are sensitive to different levels of contrast [86]. Different gamma frequencies have been theorized to reflect different perceived states produced by different stimuli [87]. Different gamma bands have also been shown to selectively register different contrasts [88]. It is possible that the mask patterns and letter fragments are being delivered through different gamma levels.

As additional evidence for the role of gamma waves, Kinsey and associates examined the role of contrast in a task that called for the recognition of objects that were placed against plain or patterned backgrounds [89]. They found synchronized gamma activity in V1/V2 on trials wherein participants recognized the figures. It is possible that the contrast differences in our study may have caused mixed signals that disrupted recognition. The mask and the letter fragments could have been delivered through two different gamma bands, with spatiotemporal integration into an incoherent message. Furthermore, the fact that clipped contour segments within the mask strips were similar in length to letter fragments may have contributed to the effectiveness of this masking condition.

Recent work by Muller et al. makes the case that traveling waves may play an integral role in the transfer of information across different regions of the brain [90]. Though many researchers have been focused on the role of ERPs in visual processing, traveling waves offer an alternative method of information transfer. Traveling waves can propagate across multiple brain regions, modulating the spiking activity among different networks. Traveling waves in the V1 and V2 regions have been evoked by feedforward stimuli and shown to be synchronized with each other, suggesting that they are allowing integration of retinotopic information across regions of the visual system [90]. This review also stated that shifts in the phase of gamma oscillations between electrodes in the V1 are consistent with the presence of waves that were traveling in the same direction. However, the function of these waves is still unclear, as is their contribution to how masking may affect recognition.

We propose that the spatial and temporal coincidence of mask strips and letter fragments interrupts the integration process and makes it difficult for subjects to form a coherent letter from the fragments. At the highest contrast levels, subjects had almost zero recognition of letters, indicating that high-contrast strips completely suppressed integration. We propose that integration is especially disrupted by the use of localized mask strips together with high mask contrast and these display conditions impair the top-down cortical processes necessary for letter recognition.
